# Identification of lncRNA expression profiles and ceRNA analysis in the spinal cord of morphine-tolerant rats

**DOI:** 10.1186/s13041-018-0365-8

**Published:** 2018-04-10

**Authors:** Jiali Shao, Jian Wang, Jiangju Huang, Chang Liu, Yundan Pan, Qulian Guo, Wangyuan Zou

**Affiliations:** Department of Anesthesiology, Xiangya Hospital, Central South University, 87 Xiangya Road, Changsha, 410008 Hunan China

**Keywords:** LncRNA, ceRNA, Morphine tolerance, Spinal cord

## Abstract

Morphine tolerance is a challenging clinical problem that limits the use of morphine in pain treatment, but the mechanisms of morphine tolerance remain unclear. Recent research indicates that long noncoding RNAs (lncRNAs) might be a novel and promising target in the pathogeneses of diseases. Therefore, we hypothesized that lncRNAs might play a role in the development of morphine tolerance. Male Sprague-Dawley rats were intrathecally injected with 10 μg morphine twice daily for 7 consecutive days. The animals were then sacrificed for lncRNA microarray tests, and the results were validated by RT-qPCR. Next, functional predictions for the differentially expressed mRNAs (DEmRNAs) were made with the Gene Ontology/Kyoto Encyclopedia of Genes and Genomes (GO/KEGG), and predictions for the differentially expressed lncRNAs (DElncRNAs) were made based on competitive endogenous RNA (ceRNA) analyses. The rats successfully developed morphine tolerance. LncRNA microarray analysis revealed that, according to the criteria of a log2 (fold change) > 1.5 and a *P*-value < 0.05, 136 lncRNAs and 278 mRNAs were differentially expressed in the morphine tolerance group (MT) compared with the normal saline group (NS). The functions of the DEmRNAs likely involve in the processes of the ion channel transport, pain transmission and immune response. The ceRNA analysis indicated that several possible interacting networks existed, including (MRAK150340, MRAK161211)/miR-219b/Tollip.Further annotations of the potential target mRNAs of the miRNAs according to the gene database suggested that the possible functions of these mRNAs primarily involved the regulation of ubiquitylation, G protein-linked receptors, and Toll-like receptors, which play roles in the development of morphine tolerance. Our findings revealed the profiles of differentially expressed lncRNAs in morphine tolerance conditions, and among these lncRNAs, some DElncRNAs might be new therapeutic targets for morphine tolerance.

## Introduction

Morphine tolerance is defined as the diminished analgesic effect and the need for a higher dose to achieve the desired analgesic effect after chronic exposure to morphine [1–4]. Over the past decades, people have attempted to elaborate the mechanisms of morphine tolerance with minimal success. The recent focus of this area is the role of non-coding RNAs (ncRNAs) [5, 6]. Among ncRNAs, some studies have reported that miRNAs are involved in the development of morphine tolerance [7, 8], including the let-7 family, miR-23b [9], miR-133b, miR-339 [10], miR-365 [11] and miR-219-5p [12]. However, the research regarding long non-coding RNAs (lncRNAs) is still in its infancy.

The sequences of lncRNAs range in size from approximately 200 nt to over 100 kb, and lncRNAs can function in novel mechanisms of the modulation of the expression of genes [13, 14]. Over the past decades, lncRNAs have been the highlight of some studies of cancer, osteoarthritis, nervous system function and development [15–17]. In pain research, recent studies have claimed that Knav2 AS [18], uc.48+ [19] and some other lncRNAs [20] might play roles in the process of the development of neuropathic pain [21, 22]. The function of lncRNAs has been illustrated as follows: lncRNAs can interact with mRNAs, bind to transcription factors, modulate chromatin remodeling, and even directly regulate the functions of target proteins. Among lncRNAs, some may act as competitive endogenous RNAs (ceRNAs) that target miRNAs [14].

The ceRNA hypothesis was proposed in 2011 with the aim of further elaborating the relationships among RNAs. Salmena et al. [23] highlighted that some RNAs act as ceRNAs that participate in mutual competition for common binding sites of target miRNAs and thus modify the functions of the target miRNAs. Recent research has indicated that ceRNA analysis might shed a light on functional predictions of the effects of lncRNAs [24].

Therefore, in the present study, we hypothesized that lncRNAs might be differentially expressed and act in direct or indirect manners during the development of morphine tolerance. To address this hypothesis, we attempted to identify the lncRNA expression profiles in the spinal cords of rats under normal and morphine tolerance conditions and to predict the possible functions of differentially expressed lncRNAs (DElncRNAs).

## Methods

### Intrathecal injection of morphine induces a chronic morphine tolerance model in rats

Adult male Sprague-Dawley rats (weight 240–260 g) were obtained from the Hunan SJA Laboratory Animal Company (Hunan, China). The rats were housed in groups and maintained on a 12/12 light-dark cycle at a room temperature of 22 ± 1 °C with food and water freely available. The experimental procedures were approved by the Animal Care and Use Committee of Central South University and conducted in strict accordance with the guidelines of the International Association for the Study of Pain [25]. To establish the rat model of morphine tolerance, rats in the morphine tolerance group (MT, *n* = 8) were intrathecally injected (i.t.) with 10 μg (1 μg/1 μl) of morphine twice daily at 08:00–09:00 am and 16:00–17:00 pm for 7 consecutive days [3, 26]. The normal saline group (NS, n = 8) was injected with equal volumes of normal saline at the same time points.

### Tail flick test

The tail flick test was used to measure thermal sensitivity. Before conducting this test, the rats were placed on the plantar surface for 15 min to adapt to the testing environment. Then, we tested one fixed point of the tail 2–3 cm from the tip using the Hargreaves apparatus (Italy, UGO Basile) [26, 27]. The results are expressed as the tail withdrawal latency (TWL), which was ultimately converted to the percent of the maximum possible effect (%MPE). The radiant index was set at 90, and the cut-off was 20 s to avoid tissue damage. The tests were conducted 30 min before and after the morning injection of morphine on days 1, 3, 5, and 7.

### Tissue collection and RNA isolation

On day 8, after the morning injection with morphine or saline, the rats were deeply anesthetized with pentobarbital sodium (1%) one hour later. Then, we decapitated the rats, collected the lumbar enlargements and placed the collected tissues into liquid nitrogen as quickly as possible for preservation. Next, we extracted the total RNA from the spinal tissues and tested the RNA quantity and quality with a NanoDrop ND-1000 Spectrophotometer (Thermo,USA) and tested the RNA integrity with agarose gel electrophoresis (2%). Subsequently, we stored the remnant RNA at − 80 °C for later use. The RNA isolation was performed by Kangcheng Bio-tech (Shanghai, China).

### Microarray assay

We employed a rat lncRNA microarray 4 × 44 k,V1.0 (Arraystar) containing approximately 9000 lncRNAs to screen the differentially expressed lncRNAs and mRNAs. The total RNAs of the MT and NS groups (*n* = 5) were hybridized with the gene chips. The RNA samples were transcribed into fluorescent cRNAs along the entire lengths of the transcripts without a 3′ bias utilizing random primers. The labeled cRNAs were hybridized to the rat lncRNA microarray. Next, the arrays were scanned with an Agilent DNA Microarray Scanner (part number G2505C). The array images were analyzed with Agilent Feature Extraction software (version 11.0.1.1). Quantile normalization and subsequent data processing were performed using the GeneSpring GX v12.1 software package (Agilent Technologies,USA). The microarray hybridization was performed by Kangcheng Bio-tech (Shanghai, China).

### Bioinformatics analysis

We used the criteria of a log2 (fold-change) > 1.5 and a *P*-value < 0.05 to screen for the deregulated RNAs. Hierarchical clustering was performed with Cluster 3.0, and the heat maps were generated in Java Treeview. The DEmRNAs were analyzed according to the pathway annotations of the Kyoto Encyclopedia of Genes and Genomes (KEGG) and Gene Ontology (GO) functional enrichment using CapitalBion. The -log_10_ (*P*-values) of the GO and pathway results are displayed in the histogram. The DElncRNAs were analyzed by ceRNA analyses, which were conducted with Arraystar’s homemade miRNA target prediction software, which is based on TargetScan and miRanda [28]. A lncRNA/miRNA/mRNA interaction network was generated to visualize the interactions using Cytoscape. The NCBI Database was used to annotate the functions of the potential target genes.

### Real-time quantitative polymerase chain reaction (RT-qPCR)

The microarray results were confirmed by RT-qPCR. The total RNAs of the MT and NS groups (*n* = 5 in each group) were reverse transcribed using random hexamer primers (Arraystar Flash RNA Labeling Kit, Arraystar) according to the manufacturer’s description. The expression levels of 18 lncRNAs (MRAK080737, MRAK159688, MRAK046606, DQ266361, XR_005988, uc.48+, uc.310-, XR_009527, XR_008662, S66184, MRAK161211, MRAK150340, XR_006440, AF196267, MRAK077287, MRAK014088, MRAK141001 and MRAK038897), as well as 12 DEmRNAs (S100a8, Batf, Ccl7, RT1-Bb, RatNP-3b, Grm2 and Mmp9, Prrx2, Asb2, Fam111a, Kcnv2 and Tmem119) were examined. GAPDH was used as the house-keeping gene. The sequences of all primers are presented in Table 1. We conducted the RT-qPCR tests with a 10 μL reaction system in a ViiA 7 Real-time qPCR System according to the manufacturer’s protocol. Melting-curve analysis was performed to monitor the specificity of the production. All experiments were replicated three times. The gene expression levels in the MT and NS groups were analyzed with the 2^−ΔΔCT^ method.

**Table 1:** The detailed information of primer sequence

Sequence name	Primer sequence	Amplicon size (bp)
GAPDH(RAT)	F:5’ GCTCTCTGCTCCTCCCTGTTCTA3’ R:5’ TGGTAACCAGGCGTCCGATA3’	124
MRAK161211	F:5’CTGACCCCAAAGTTTCACATCT3’ R:5’CCAGGAGAGGTGTTCCAAGTAA3’	63
MRAK038897	F:5’TGGCAAGAATACCAAAGAGC3’ R:5’CACAGCAAGATGTAATGCACAG3’	132
MRAK014088	F:5’GTGTCTATTTCTGGGAGTCTGTGC3’ R:5’GCCATTGGTAAGAGAATTAAGCAG3’	102
MRAK080737	F:5’GTGCCAGACCCCAAGGTAAA3’ R:5’GAGACAATAATGGAGCCGCC3’	105
MRAK159688	F:5’GTACTGTAGCTCTTCAGCGTCC3’ R:5’GTCAGAACATTCAGACCACCTC3’	78
MRAK046606	F:5’GCCAGCATCTCCTACTCACA3’ R:5’TGGACTACGGACTACAGTTTACC3’	80
MRAK150340	F:5’ACAGAGTAGGGCAGTCGCAG3’ R:5’GGTTGTGGACCATAGAAGAGTTG3’	205
DQ266361	F:5’TGTGGTTAAATCCCCATGC3’ R:5’CTTCTCCAAGTCACATCTGCTC3’	63
XR_006440	F:5’GGAGCATCAAATCGAAAGC3’ R:5’ACTCGGATCGTCTCAAGGAC3’	162
XR_005988	F:5’TGTGACACCACTGAGACCCTT3’ R:5’TGGCCCTCCACACTTTACGA3’	101
uc.48+	F:5’ AAATGCAAACTGGATGAGGA 3′ R:5’ GTTAACACTGTATGTAATTAGGG 3’	279
uc.310-	F:5’ CTAATCAAAAACTGACAGCAAGA 3′ R:5’ GATCTTTCTTAAGCAGAATTTGG 3’	128
XR_009527	F:5’ CCAAGGCCCGTATTGAGATTA 3′ R:5’ AGGGTCCAATGTGCCACGA 3’	116
XR_008662	F:5’ TAATGAGGAAGATGAGAATGGC 3′ R:5’ CCAGATAGGCTTCGTCTTATTC 3’	103
S66184	F:5’ TCTTCACATTACCATTACGAGGA 3′ R:5’ CATCGGAATGATTTTGCTGTGT 3’	51
AF196267	F:5’ GCCATCAATTTTCTCTTGACTG 3′ R:5’ TGAAGGGTCAGTTTGAAGCA 3’	135
MRAK077287	F:5’ GCTAATAATTCCTACCAGCAAA 3′ R:5’ ACCTCACACCCAGTCTCTACAT 3’	151
MRAK141001	F:5’ CTTCCCTACCAGTCTATTGAGTG 3′ R:5’ ACGCTCCACTACAAAATCAGTT 3’	87
S100a8	F:5’GGGAATCACCATGCCCTCTA3′ R:5’GCCCACCCTTATCACCAACA3’	168
Batf	F:5’GAGGACCTGGAGAAACAGAATG3’ R:5’GCTCAGCACCGATGTGAAGTA3’	87
Ccl7	F:5’GCTGCTATGTCAAGAAACAAAAGA3’ R:5’TGATGGGCTTCAGCACAGACT3’	136
RT1-Bb	F:5’GCCCTCAACCACCACAACTT3’ R:5’GGTCCAGTCCCCGTTCCTAAT3’	141
Prrx2	F:5’AAGAAGAAGCAGCGTCGGA3’ R:5’CAAAGGCGTCAGGGTAGTGT3’	97
Asb2	F:5’TGCTTTTCCTGCCTGTATGG3’ R:5’CGACAGGAACTCACAGAACTGC3’	120
RatNP-3b	F:5’ CATACGCCAAAGTCTGAAACC 3′ R:5’ AGCAGTGCCTTTATCCCCTC 3’	168
Grm2	F:5’ CCCGGAGAACTTCAACGAA 3′ R:5’ GGCTGGAAAAGGATGATGTG 3’	207
Mmp9	F:5’ CCCACTTACTTTGGAAACG 3′ R:5’ GAAGATGAATGGAAATACGC 3’	228
Fam111a	F:5’ GACTATTTCTCTCAGGTTCCCA 3′ R:5’ GTGCTGCATACAAGCTACTTGT 3’	256
Kcnv2	F:5’ GGGCTGCGGTAAGCATCTCT 3′ R:5’ TTGAGAATAATCCCAAAAGCGA 3’	106
Tmem119	F:5’ AGACAGTCGAACGGTCTAACAG 3′ R:5’ TCACAAGTAGCAGCAGAGACAG 3’	127

### Statistical analysis

All data were presented as mean ± s.e.m. The statistical significance of differences between groups was analyzed with two-way repeated-measures of ANOVA followed by Bonferroni test or with Student’s *t*-test. *P* values less than 0.05 were considered statistically significant.

## Results

### Construction of the rat morphine tolerance model

**Fig. 1:**
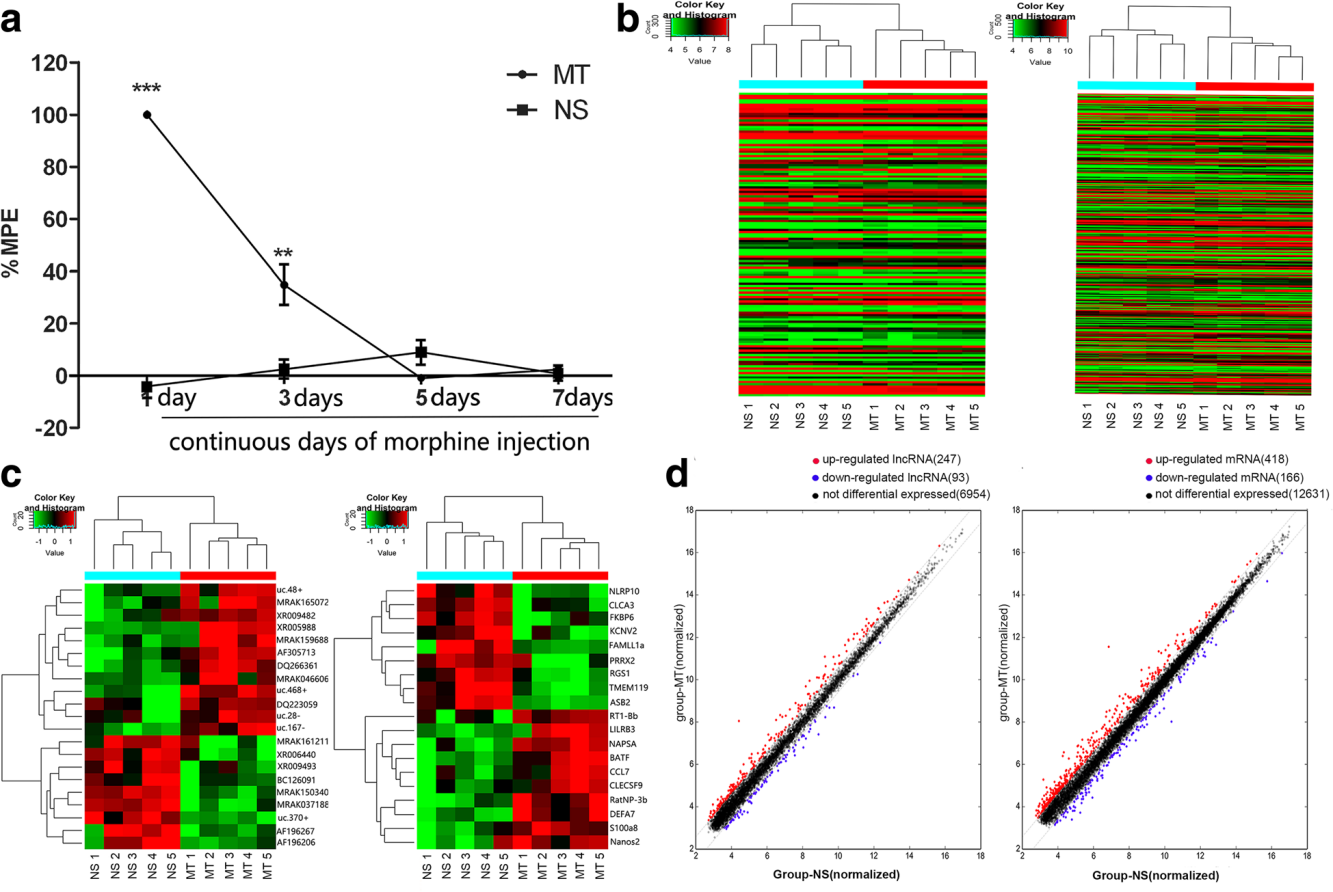
(**a)** Continuous injection of morphine for 7 days induced the formation of morphine analgesic tolerance. The data are expressed as the mean ± s.e.m. (n = 8 in each group). **P < 0.01, ***P < 0.001, Two-way repeated-measures of ANOVA followed by Bonferroni test. Ten samples (n = 5 in each group) were subjected to microarray analysis. **b**-**c** The entire and partial hierarchical clusterings of the lncRNAs and mRNAs, respectively; the up- and down-regulated genes are colored in red and green, respectively. **d** Scatter plot displaying the lncRNAs and mRNAs that exhibited expression differences between the MT and NS groups that exceeded 1.5-fold

The tail flick test data revealed that there were no significant changes in the thermal sensitivities of NS group (*n* = 8 in each group). However, comparisons between the two groups revealed that, on day 1 post-injection, the %MPE of the MT group (n = 8 in each group) was significantly higher than that of the NS group (*P*<0.05). On day 3 post-injection, the % MPE of the MT group began to decline but remained higher than that of the NS group (*P*<0.05). On day 5 post-injection, the %MPEs did not significantly differ between the two groups (*P*>0.05), and this state continued to day 7 (Fig. 1a), which suggested that a stable morphine tolerance model had been established.

### Overview of the lncRNA and mRNA expression profiles

First, we created an overview of the lncRNA and mRNA expression profiles using a using scatter plot, which revealed that large numbers of lncRNAs and mRNAs were differentially expressed between the MT and NS groups (*n* = 5; Fig. 1d). Next, hierarchical cluster analyses of all of the lncRNAs and mRNAs was applied and revealed that the 5 NS and 5 MT samples clustered independently, and the results also indicated high degrees of consistency in both the NS and MT groups (Fig. 1b, c). All of the microarray results have been uploaded to the GEO database (GSE110115).

### Differentially expressed lncRNAs and mRNAs in morphine tolerance

According to the criteria of a log2 (fold change) > 1.5 and a *P*-value< 0.05, the microarray data identified 136 lncRNAs, including 84 up-regulated and 52 down-regulated lncRNAs, which were significantly altered in the MT group compared with the NS group. The lncRNAs that exhibited the greatest up-regulations were XR_005988, DQ266361, and MRAK046606 with XR_005988 exhibiting the largest up-regulation [log2 (fold change) =12.4243]. The lncRNAs that exhibited the greatest down-regulations were AF196267, XR_009493, and MRAK150340 with AF196267 exhibiting the largest down-regulation [log2 (fold change) =2.2025]. Detailed information, including the top 40 up-regulated and top 40 down-regulated lncRNAs, is provided in Table 2.

**Table 2:** The detailed information of top 40 up-regulated and 40 down-regulated lncRNAs

Up-regulated lncRNAs	Fold change(MT/NS)	P-value	Down-regulated lncRNAs	Fold change(MT/NS)	*P*-value
XR_005988	12.34156	0.003685	AF196267	2.202529	0.012181
DQ266361	2.486278	0.000882	XR_009493	2.115125	0.019025
MRAK046606	2.367485	0.020344	MRAK150340	2.060765	0.000308
uc.167-	2.199281	0.002987	MRAK037188	1.956905	0.000066
uc.468+	2.137996	0.000249	XR_006440	1.952104	0.003301
XR_009482	2.065426	0.022224	AF196206	1.938910	0.015500
AF305713	2.052460	0.001121	BC126091	1.906750	0.020304
MRAK165072	2.020835	0.015095	uc.370+	1.900405	0.000477
MRAK159688	2.009110	0.008496	MRAK156916	1.879795	0.013478
uc.28-	1.957873	0.033513	XR_008800	1.872603	0.022847
DQ223059	1.911577	0.043779	MRAK077287	1.816863	0.000017
uc.48+	1.897821	0.005311	MRAK014088	1.773360	0.000324
XR_006726	1.887222	0.008923	MRAK161211	1.765669	0.014718
XR_009483	1.883845	0.020077	BC158785	1.742858	0.005583
MRAK013672	1.849562	0.027735	MRuc009dux	1.721422	0.014515
MRAK018927	1.848401	0.025469	MRAK135122	1.718807	0.009117
uc.482-	1.827252	0.000618	MRAK141001	1.690469	0.000352
MRAK138235	1.824250	0.005928	MRuc007nwi	1.688666	0.043028
uc.156-	1.810205	0.037932	uc.264-	1.687839	0.023742
XR_008353	1.802395	0.008201	EF088428	1.673345	0.001656
uc.462+	1.791152	0.001958	uc.363+	1.672870	0.011181
MRAK134839	1.770980	0.033987	BC169026	1.665411	0.007442
MRAK054291	1.760502	0.037076	MRAK008891	1.659204	0.018204
BC093392	1.758918	0.030404	MRAK135686	1.656007	0.005067
MRuc007cgx	1.756746	0.023148	MRAK051195	1.625373	0.000267
MRAK050995	1.742487	0.032134	BC097960	1.619298	0.003837
XR_009527	1.742256	0.022408	MRAK169397	1.617138	0.030259
uc.395-	1.733383	0.035051	MRAK046121	1.614043	0.005023
XR_005532	1.732123	0.042440	MRAK080238	1.594013	0.000840
uc.158-	1.723747	0.010144	BC061963	1.592978	0.002208
MRuc007jeg	1.722591	0.006501	MRAK013677	1.592863	0.000261
XR_008662	1.716865	0.020951	XR_009137	1.575140	0.014863
XR_008674	1.695926	0.047672	BC086373	1.561696	0.008523
S66184	1.680145	0.026505	MRAK083715	1.557152	0.004611
M81783	1.670161	0.011349	MRAK038897	1.555085	0.007252
MRAK083472	1.662885	0.008172	MRAK041309	1.554516	0.000067
XR_009489	1.660491	0.002298	MRAK147844	1.551990	0.000730
XR_008266	1.654757	0.012200	MRAK051810	1.549681	0.022164
uc.310-	1.653784	0.013204	uc.185+	1.545991	0.024361
uc.463-	1.651362	0.021613	BC079474	1.544677	0.000147

Regarding the DEmRNAs, there were 278 genes (176 up-regulated and 102 down-regulated) whose changes met the criteria. These DEmRNAs contained many genes that are known to be involved in pain processing, including Ccl7, Batf, S100a8, Kcnv2, Rgs1, Prrx2, Mmp9, etc. Detailed information about the top 30 up-regulated and top 30 down-regulated mRNAs is listed in Table 3.

**Table 3:** The detailed information of top 30 up-regulated and 30 down-regulated mRNAs

Gene symbol	Description	Fold change(MT/NS)	*P*-value
Up-regulated genes			
RT1-Bb	RT1 class II, locus Bb	26.0600137	0.03123893
RatNP-3b	defensin RatNP-3 precursor	4.6945682	0.00237615
Lilrb3	leukocyte immunoglobulin-like receptor, subfamily B (with TM and ITIM domains), member 3	4.3359795	0.00815228
Defa7	alpha-defensin 7	4.2552547	0.00156229
Clecsf9	macrophage-inducible C-type lectin	3.4622832	0.01864872
Ccl7	chemokine (C-C motif) ligand 7	3.3728046	0.01483951
Sele	selectin, endothelial cell	3.3632425	0.03028204
Slpi	secretory leukocyte peptidase inhibitor	3.2118152	0.00024306
Lilrc2	leukocyte immunoglobulin-like receptor	3.1471364	0.01375120
V1rj4	vomeronasal 1 receptor, J4	2.9047312	0.03220865
Gja5	gap junction membrane channel protein alpha 5	2.8707048	0.02230140
Mmp9	matrix metallopeptidase 9	2.832538	0.02991875
Batf	basic leucine zipper transcription factor	2.7432184	0.00125327
S100a8	S100 calcium binding protein A8 (calgranulin A)	2.7009035	0.00561932
Birc3	baculoviral IAP repeat-containing 3	2.6968995	0.01058921
Bcl3	B-cell CLL/lymphoma 3	2.6847827	0.00419171
Ccr1	chemokine (C-C motif) receptor 1	2.5775393	0.00007731
Olr463	olfactory receptor 463 (predicted)	2.5772986	0.01425058
Np4	defensin NP-4 precursor	2.4883044	0.00271115
Grm2	glutamate receptor, metabotropic 2	2.4429944	0.00113554
Slpil2	antileukoproteinase-like 2	2.4380328	0.00066484
Cnn1	calponin 1	2.4269788	0.02918954
Olr139	olfactory receptor Olr139	2.4268332	0.01332394
Obp3	alpha-2u globulin PGCL4	2.3723118	0.03995990
Crisp4	cysteine-rich secretory protein 4	2.3633549	0.00810051
Olr1454_predicted	olfactory receptor 1454 (predicted)	2.3350625	0.00247670
Napsa	napsin A aspartic peptidase	2.2770041	0.00035275
Olr1374_predicted	olfactory receptor 1374 (predicted)	2.2313479	0.00040213
LOC497796	Ly49 inhibitory receptor-like	2.2302475	0.00022859
Slpil3	antileukoproteinase-like 3	2.2153032	0.00045886
Down-regulated genes			
Fam111a	hypothetical protein LOC499322	5.1287218	0.02433528
Kcnv2	“potassium channel, subfamily V, member 2”	2.8837136	0.00586212
Fkbp6	FK506 binding protein 6	2.8648794	0.00576975
Nlrp10	“NLR family, pyrin domain containing 10”	2.6101210	0.00386452
Rgs1	regulator of G-protein signaling 1	2.4933037	0.00327101
Ly49i8	Ly49 inhibitory receptor 8	2.3732515	0.01051908
Tmem119	transmembrane protein 119	2.3374630	0.00064129
Cldn14	“*Rattus norvegicus* claudin 14”	2.3184480	0.00751794
Dyrk1a	dual-specificity tyrosine-(Y)-phosphorylation regulated kinase 1A	2.2472669	0.00077705
Ckmt2	sarcomeric mitochondrial creatine kinase	2.2142101	0.00051314
Asb2	ankyrin repeat and SOCS box-containing protein 2	2.1709796	0.00039719
Art2b	ADP-ribosyltransferase 2b	2.1680042	0.00124959
LOC498335	similar to Small inducible cytokine B13 precursor (CXCL13)	2.1501415	0.00012158
Prrx2	paired related homeobox 2	2.1294428	0.01452534
LOC364773	aldo-keto reductase family 1, member C12	2.1023343	0.00302323
Cdkn2b	cyclin-dependent kinase inhibitor 2B (p15, inhibits CDK4)	2.0882944	0.00336651
Cd22	CD22 molecule	2.0855279	0.01215534
Nhlrc2	NHL repeat containing 2	2.0426710	0.00054605
Fcrls	Fc receptor-like S, scavenger receptor	2.0407116	0.00627688
Clca3	chloride channel calcium activated 3	2.0188429	0.01580260
Dntt	deoxynucleotidyltransferase, terminal	2.0019106	0.00547055
Grap2	GRB2-related adaptor protein 2	1.9900082	0.00968363
Thrsp	thyroid hormone responsive protein	1.9814822	0.02740488
Plek2	pleckstrin 2	1.9662998	0.00879852
Alox12	arachidonate 12-lipoxygenase	1.9121487	0.00631083
Tnfsf4	tumor necrosis factor (ligand) superfamily, member 4	1.8804698	0.00229007
Mpzl2	myelin protein zero-like 2	1.8783013	0.00003750
Prkag3	protein kinase, AMP-activated, gamma 3	1.8643095	0.00970172
Nr0b2	nuclear receptor subfamily 0, group B, member 2	1.8348501	0.00251892
Traf3ip3	TRAF3 interacting protein 3	1.8326037	0.02787730

### Validation of the lncRNA and mRNA expressions

**Fig. 2:**
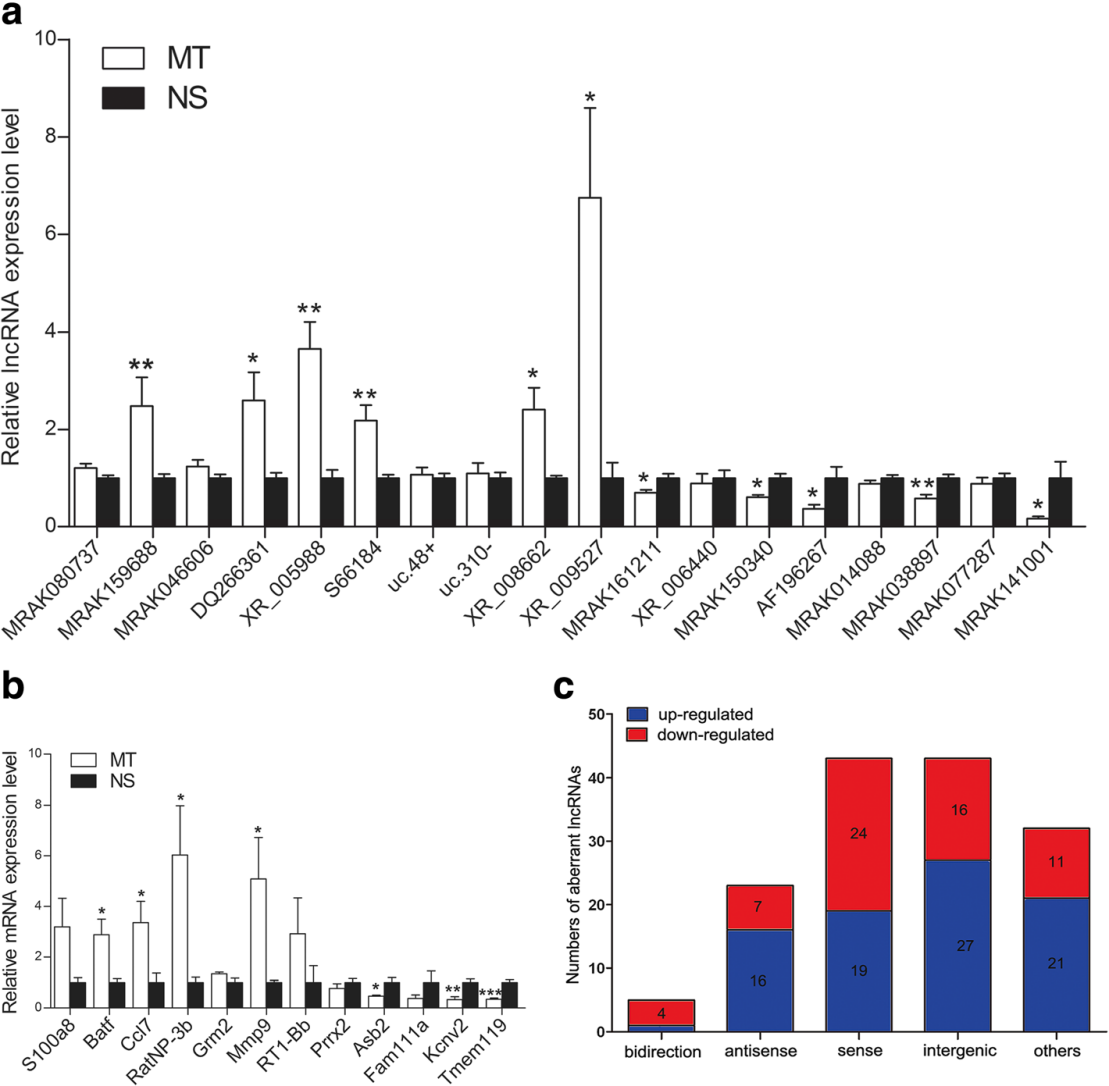
RT-qPCR validation of eighteen deregulated lncRNAs (**a**) and twelve deregulated mRNAs (**b**) in the lumbar enlargements of both groups. Student’s *t*-test. *P < 0.05, **P < 0.01, ***P < 0.001. Distribution of the various types of DElncRNAs. **c** Five classes (bidirectional lncRNAs, antisense lncRNAs, sense lncRNAs, intergenic lncRNAs and the other lncRNAs) were analyzed

To validate the reliability of the microarray results, 18 DElncRNAs and 12 DEmRNAs were selected and validated by RT-qPCR. The data shown that 11 of 18 selected DElncRNAs (XR_005988, DQ266361, MRAK159688, XR_008662, XR_009527, S66184, MRAK150340, MRAK161211, MRAK038897, AF196267 and MRAK141001) and 7 of 12 selected DEmRNAs (Batf, Ccl7, RatNP-3b, Mmp9, Kcnv2, Tmem119 and Asb2) exhibited the same trends in altered expressions and the same significant differences in the microarray and RT-qPCR analyses. On the other hand, the other altered mRNAs and lncRNAs exhibited the trends in changes, but the differences between the two groups were not significant, possibly due to the small sample size (Fig. 2a, b).

### Class distributions of the DElncRNAs

The examined lncRNAs were categorized into five groups according to their associations with coding genes: intergenic lncRNAs, antisense overlap lncRNAs, sense overlap lncRNAs, bidirectional lncRNAs, and other. One of the mechanisms by which lncRNAs act is through the interplay with adjacent coding genes [20, 29]; therefore, it was important to classify the locations of the lncRNAs. Our data revealed that, among these DElncRNAs, intergenic lncRNAs and sense overlap lncRNAs accounted for the majority, and only five lncRNAs belonged to the bidirectional category. The concrete data are presented in Fig. 2c.

### Functional predictions for the DEmRNAs in the morphine-tolerant rats

**Fig. 3:**
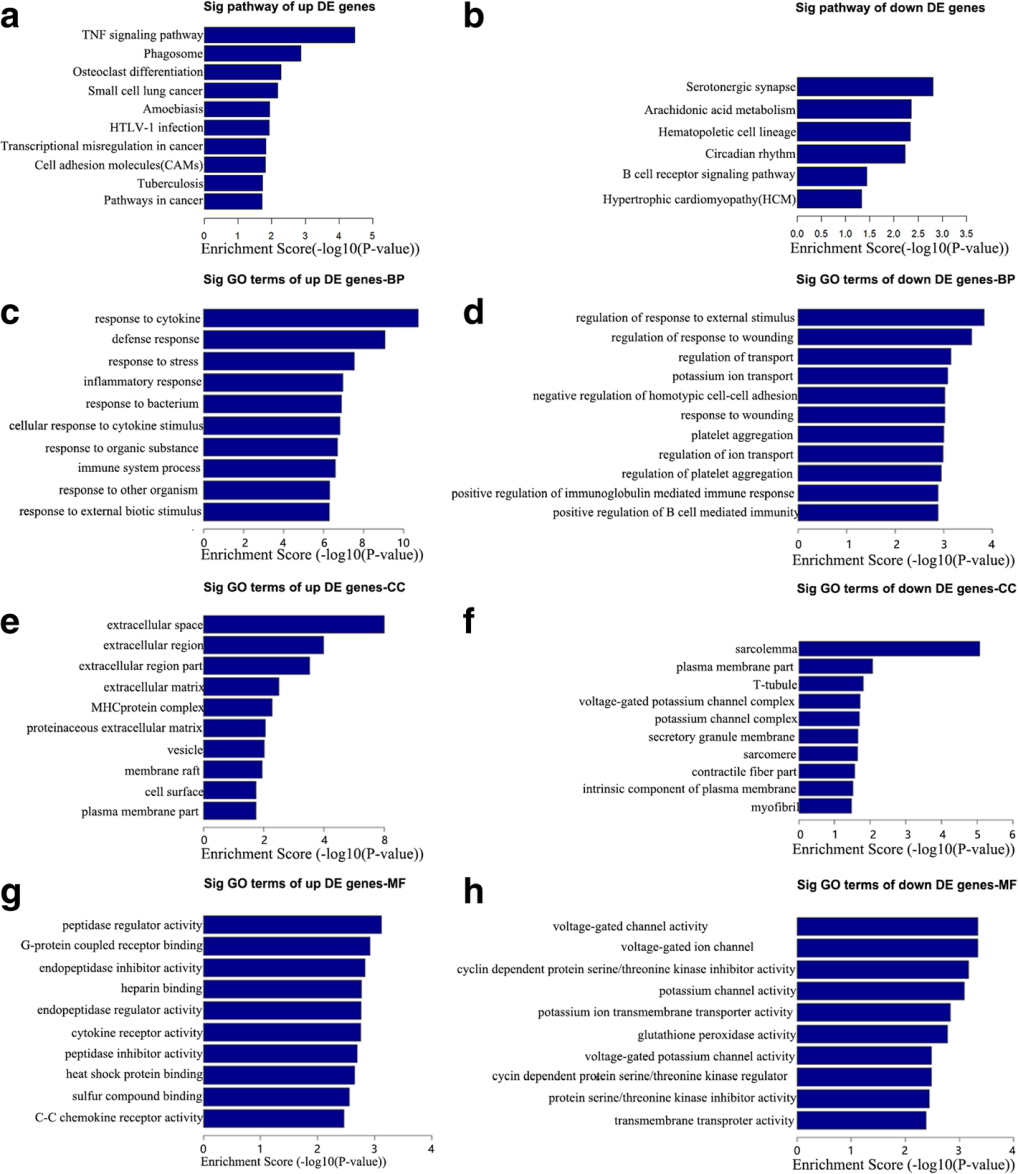
Pathway analyses of the 176 up-regulated and 102 down-regulated mRNAs with fold changes > 1.5. **a** The significant pathways of the up-regulated genes in the MT group. **b** The significant pathways of the down-regulated genes in the MT group. The biological functions of the differentially expressed mRNAs with fold changes > 1.5 are listed. The significant biological processes (**c**) cellular components (**e**) and molecular functions (**g**) of the up-regulated mRNAs. The significant biological processes (**d**), cellular components (**f**) and molecular functions (**h**) of the down-regulated mRNAs

To explore the molecular functions of the DEmRNAs in morphine tolerance conditions, we further performed GO and pathway analyses genes that differentially regulated in the MT and NS groups. The pathway analyses indicated that the most significantly enriched pathways of the up-regulated genes included the TNF metabolic pathway and phagocytic processes, and the down-regulated genes were involved in synaptic vesicle activity, the arachidonic acid metabolic pathway, etc. (Fig. 3a, b). The GO results revealed that the most significantly enriched molecular functions of the up-regulated genes in the MT group were peptidase activity, G-protein-coupled receptor activity, and biological processes concentrated on the cytokine response, defense and the immune response. The cell components primarily belonged to extracellular domains and intercellular domains. The most significantly enriched molecular functions of the down-regulated genes in the MT group were voltage-gated channel activity, ion transmembrane activity, and biological processes concentrated on potassium ion transport. The cell components were associated with the sarcolemma, cytoplasmic membrane, and ion channel complexes (Fig. 3c-h).

### Functional predictions of the DElncRNAs lncRNA/miRNA/mRNA interactions

**Fig. 4:**
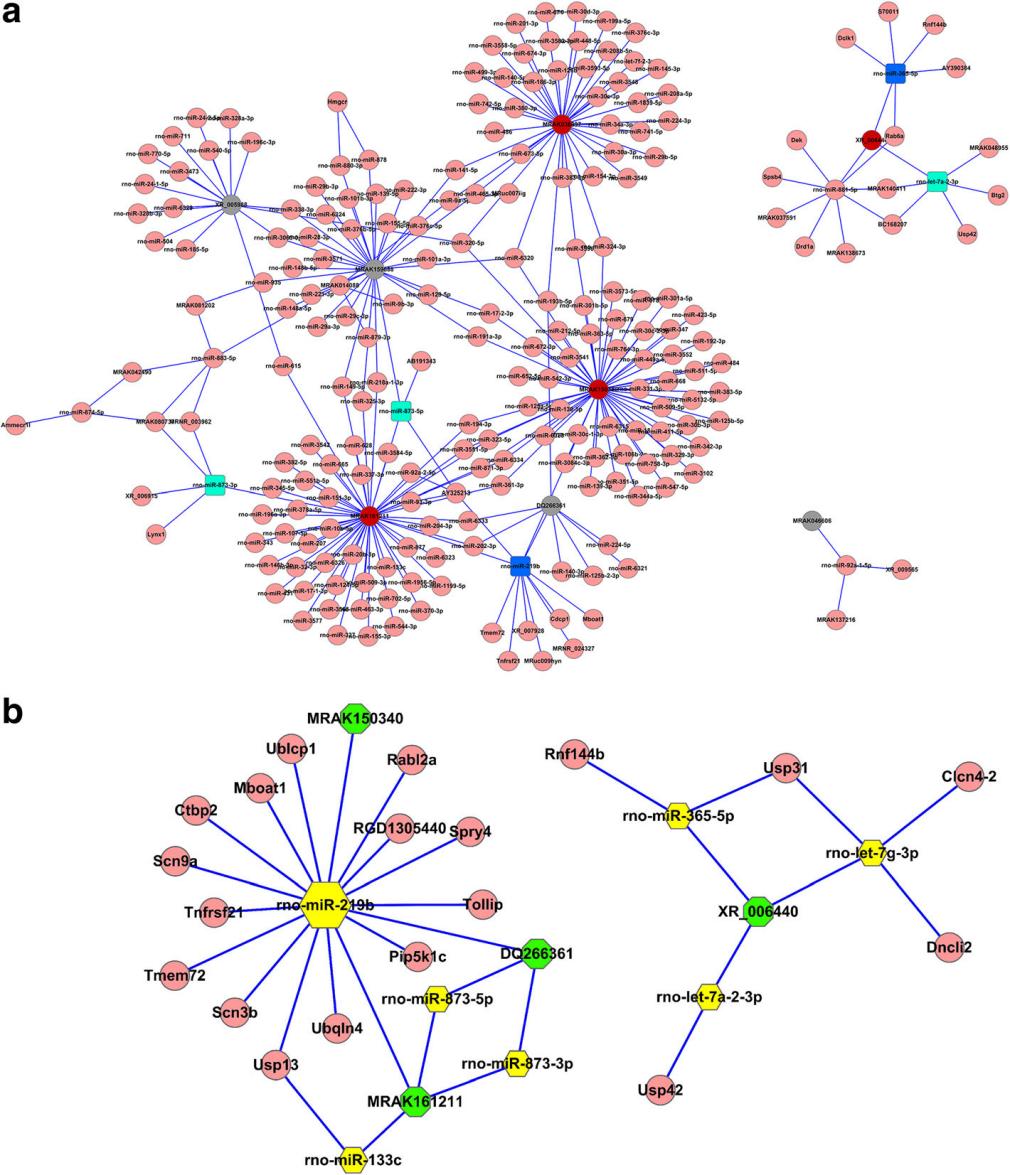
ceRNA analyses indicated the potential lncRNA/miRNA/mRNA interactions. **a** The potential binding target miRNAs of the verified lncRNAs. The red nodes mean down-regulated lncRNAs, the gray nodes mean up-regulated lncRNAs, the blue squares mean down-regulated miRNAs we are interested in, the green squares mean up-regulated miRNA we are interested in, and the pink nodes mean the other miRNAs and mRNAs. **b** The lncRNA/miRNA/mRNA networks that we are interested in are displayed. The green nodes represent lncRNAs, the yellow pentagons represent miRNAs and the pink nodes represent mRNAs we forecasted

We performed coding-noncoding gene co-expression (CNC) analysis, but we found no DEmRNA-associated DElncRNAs, which meant that we were unable to make forecasts about the functions of the DElncRNAs according to the related DEmRNAs. However, ceRNA analysis allowed us to predict the possible functions of the DElncRNAs regardless of their adjacent coding genes. According to the ceRNA analysis, we obtained an overview of the potential lncRNA**/**miRNA/mRNA interactions (Fig. 4a), and we then further identified several promising networks of lncRNA/miRNA/mRNA interactions (Fig. 4b), which included (MRAK161211, MRAK150340)/miR-219b/Tollip and XR_006440/(miR-365, let7)/(Usp31, Usp42, Clcn4–2) networks, and we used these networks to create functional annotations of the predicted target mRNAs by searching the Gene database. The results indicated that the predicted target mRNAs mainly functioned in the process of ubiquitinylation, the GRCP and TLR signaling pathways, and the modulations of transcription, translation and post-translational modification, and these functions might constitute the foundation of morphine tolerance.

## Discussion

In the present study, we detected 136 DElncRNAs and 278 DEmRNAs overall and we found that compared with normal rats, DElncRNAs and DEmRNAs were present in the spinal cords of morphine-tolerant rats; GO and KEGG pathway analyses revealed that the potential functions of the DEmRNAs may be concentrated on the processes that are thought be involved in the formation of morphine tolerance; and the ceRNA analysis identified several potential lncRNA/miRNA/mRNA interaction networks that might modulate the development of morphine tolerance.

To identify as many DElncRNAs and DEmRNAs candidates as possible, we set the criteria at a log2 (fold change) > 1.5 and a *P*-value < 0.05 [30]. Next, we considered several criteria to select lncRNAs and mRNAs from our microarray data for validation. Firstly, we attempted to select the relevant candidates that might be related to our previous studies of morphine tolerance and miRNAs [11, 12]. Secondly, for validation, we chose the lncRNA candidates that exhibited higher fold changes and greater raw expression intensities and had adjacent mRNA that was related to morphine tolerance.

Among the DElncRNAs that we detected, XR_005988 exhibited the most significant up-regulation and was classified as a long intergenic non-coding RNA (lincRNA). Although we obtained no information about XR_005988 or its associated genes through searches of all types of gene databases, it is well known that lncRNAs account for the main portion of lncRNAs and exhibit the most substantial biological functions [31], which indicates that XR_005988 is still a promising lncRNA molecule for further study. Additionally, XR_005988 might bind to some miRNAs according to the ceRNA analysis, which indicates its potential action as a ceRNA. Of course, additional in vivo and in vitro functional research is needed.

MRAK-159688, which was another up-regulated lncRNA identified in our study, is associated with the Fos gene and was named the Fos downstream transcript (FosDT) in a report from Mehta et al. [32] Mehta et al. reported that, in an ischemia/reperfusion model, FosDT interacts with the chromatin-modifying proteins Sin3a and co-repressor of the transcription factor REST (coREST) and subsequently represses REST-downstream genes. Moreover, other researchers have indicated that MOR is one of the downstream targets of REST and is negatively modulated by REST in specific neuronal cells [33, 34]. Therefore, the dysregulated MRAK-159688 might be involved in the development of morphine tolerance through interactions with REST.

Since the ceRNA hypothesis was proposed, it has been verified in some tumor diseases. For example, the FER1L4/miRNA106a-5p/PTEN pathway constitutes a novel regulatory pathway that is involved in the occurrence and progression of gastric cancer [35]. Currently, ceRNA analysis is a novel method for predicting the functions of lncRNAs. In our study, we found that several possible interacting pathways among ceRNAs exist, including the following: MRAK161211, MRAK150340/miR-219b/mRNAs (e.g., Tollip, and Ubqln4); XR_006440/miR-365, let7/mRNAs (e.g., Usp31, Usp42, Clcn4–2); and MRAK161211/miR-133/Usp13.

In our previous study, We have confirmed that miR-219-5p can attenuate morphine tolerance by targeting CaMKIIγ [12]. Furthermore, other researchers have also reported that miR-219 is down-regulated following the continuous application of morphine and can regulate NMDA receptor signaling [36]. Whereas in the present study, we predicted that miR-219b, rather than miR-219-5p, would exert this function. Recent research about miR-219 has mainly concentrated on the functions of miR-219-5p, and the other isoform, i.e., miR-219b, has not been studied in detail. However, on the one hand, we identified miR-219-5p and miR-219b, which have both previously been reported to function in the suppression of the proliferation, migration and invasion of cells [37, 38]. On the other hand, when we forecasted the downstream targets of miR-219b, we found that their functions were mainly related to the ubiquitination process, G-protein-coupled receptors, alterations in ion channels, and Toll-like receptor signaling pathway regulation, and these function are all involved in the mechanisms of morphine tolerance. Therefore, given its possible target mRNAs, miR-219b is likely to be a new target related to morphine tolerance.

Toll-interacting protein (Tollip) is a potential downstream target of miR-219b and can modulate the expression of the Toll-like receptor (TLR) via its action as an endogenous inhibitor and regulate the IL-1β-induced activation of NF-κB; thus, miR-219b plays an inhibitory role in inflammatory signaling [39, 40]. Previous studies have demonstrated that the TLR-mediated activation of glial cells and TLR4-mediated NF-κB activation might influence the development of morphine tolerance [41, 42]. Therefore, further study is required and we presumed that the MRAK150340, MRAK161211 (i.e., down-regulated lncRNAs)/miRNA-219b/Tollip interaction network potentially functions in morphine tolerance.

On the one hand, previous studies have suggested that let-7 can suppress the expression of MOR [43], and our previous results suggest miR-365 can modulate morphine tolerance by targeting the beta-arrestin 2 protein [11]. On the other hand, Law et al [44] reported that opioid receptor agonists (such as morphine) promote the ubiquitylation of scaffold proteins and thereby change the expression pattern of the receptor signaling pathway. In the present study, the potential downstream genes that we predicted, i.e., Usp31 and Usp42, are both ubiquitylation-related genes. Therefore, the XR_006440/(let7, miR-365)/(Usp31, Usp42) pathway is likely to be responsible for the formation of morphine tolerance. Moreover, there are other interested candidates other than the validated lncRNAs and mRNAs, and further studies will be needed.

In summary, although our study is preliminary and lacks additional functional experiments, our research has revealed that hundreds of lncRNAs, especially XR_005988 and MRAK159688, are differentially expressed in the spinal cords of morphine-tolerant rats. Further ceRNA analysis revealed that several possible lncRNA/miRNA/mRNA interaction networks exist, and among these networks, the (MRAK150340, MRAK161211)/miR-219b/Tollip network holds the most potential for further studies.

## Abbreviations

ceRNA: competitive endogenous
DElncRNA: Differentially expressed lncRNA
DEmRNA: Differentially expressed mRNA
GO: Gene Ontology
KEGG: Kyoto Encyclopedia of Genes and Genomes
lncRNA: Long noncoding RNA
MOR: μ-opioid receptor
MT: Morphine tolerance
NCBI: National Center for Biotechnology Information
ncRNA: Noncoding RNA
NS: Normal saline
Tollip: Toll-interacting protein
